# Altered Immune Responses in Rhesus Macaques Co-Infected with SIV and *Plasmodium cynomolgi*: An Animal Model for Coincident AIDS and Relapsing Malaria

**DOI:** 10.1371/journal.pone.0007139

**Published:** 2009-09-23

**Authors:** Jeffrey W. Koehler, Michael Bolton, Amanda Rollins, Kirsten Snook, Eileen deHaro, Elizabeth Henson, Linda Rogers, Louis N. Martin, Donald J. Krogstad, Mark A. James, Janet Rice, Billie Davison, Ronald S. Veazey, Ramesh Prabhu, Angela M. Amedee, Robert F. Garry, Frank B. Cogswell

**Affiliations:** 1 Department of Microbiology and Immunology, Tulane University School of Medicine, New Orleans, Louisiana, United States of America; 2 Department of Bacteriology and Parasitology, Tulane National Primate Research Center, Covington, Louisiana, United States of America; 3 Department of Microbiology, Tulane National Primate Research Center, Covington, Louisiana, United States of America; 4 Department of Comparative Pathology, Tulane National Primate Research Center, Covington, Louisiana, United States of America; 5 Department of Tropical Medicine, Tulane University School of Public Health and Tropical Medicine, New Orleans, Louisiana, United States of America; 6 Department of Epidemiology, Tulane University School of Public Health and Tropical Medicine, New Orleans, Louisiana, United States of America; 7 Department of Microbiology, Immunology, and Parasitology, Louisiana State University Health Sciences Center, New Orleans, Louisiana, United States of America; Comprehensive AIDS Research Center, China

## Abstract

**Background:**

Dual epidemics of the malaria parasite *Plasmodium* and HIV-1 in sub-Saharan Africa and Asia present a significant risk for co-infection in these overlapping endemic regions. Recent studies of HIV/*Plasmodium falciparum* co-infection have reported significant interactions of these pathogens, including more rapid CD4+ T cell loss, increased viral load, increased immunosuppression, and increased episodes of clinical malaria. Here, we describe a novel rhesus macaque model for co-infection that supports and expands upon findings in human co-infection studies and can be used to identify interactions between these two pathogens.

**Methodology/Principal Findings:**

Five rhesus macaques were infected with *P. cynomolgi* and, following three parasite relapses, with SIV. Compared to macaques infected with SIV alone, co-infected animals had, as a group, decreased survival time and more rapid declines in markers for SIV progression, including peripheral CD4+ T cells and CD4+/CD8+ T cell ratios. The naïve CD4+ T cell pool of the co-infected animals was depleted more rapidly than animals infected with SIV alone. The co-infected animals also failed to generate proliferative responses to parasitemia by CD4+ and CD8+ T cells as well as B cells while also having a less robust anti-parasite and altered anti-SIV antibody response.

**Conclusions/Significance:**

These data suggest that infection with both SIV and *Plasmodium* enhances SIV-induced disease progression and impairs the anti-*Plasmodium* immune response. These data support findings in HIV/*Plasmodium* co-infection studies. This animal model can be used to further define impacts of lentivirus and *Plasmodium* co-infection and guide public health and therapeutic interventions.

## Introduction

Human immunodeficiency virus type 1 (HIV) and *Plasmodium*, the malaria parasite, each present immense disease burdens worldwide. Over 33 million people are infected with HIV resulting in 3 million deaths annually [1], and each year there are approximately 500 million episodes of clinical malaria leading to over 1 million deaths [2]. With large overlapping endemic regions and the large numbers of people infected, especially in Sub-Saharan Africa and Southeast Asia, there is high risk of co-infection with HIV and *Plasmodium*. Recent studies on *P. falciparum* and HIV co-infection have found significant interactions between these two pathogens. HIV infection appears to increase the risk of both malaria parasite infection and the development of clinical malaria [3]–[10], and this risk rises with HIV-induced immunosuppression [4], [6]–[9]. *P. falciparum* infection also appears to have an impact on the HIV infection through a transient increase in HIV viral load [11], [12] and a more rapid CD4+ T cell decline [13]. A recent report by Abu-Raddad and colleagues described dual HIV/*Plasmodium* infection in Kisumu, Kenya (pop 200,000) enhanced the spread of both pathogens, leading to an increase of, from 1980 to 2005, approximately one million episodes of clinical malaria and 8,500 cases of HIV infection [10]. Clearly, co-infection has an immense impact on both diseases' progression and on the associated public health response. A well-defined animal model could help guide such a response as well as help gain insight on the pathogenesis of both infections.

Simian immunodeficiency virus (SIV) infection in rhesus macaques has been widely used to model events during HIV infection [14]–[18]. During the acute HIV/SIV infection, there is a rapid and nearly complete depletion of the mucosal CD4+ T cell population [17] followed by a gradual decline in the peripheral CD4+ T cell population [16]. An attempt is made to replenish the lost memory CD4+ T cell pool by the naïve T cell population among conventional progressors, leading to a depletion of the naïve T cell pool and, ultimately, immune system collapse and disease [15]. *P. vivax*, a relapsing malaria parasite, causes disease during its clinically relevant blood stage through the development of a strong pro-inflammatory immune response and erythrocyte loss. Clinical immunity, which develops following multiple parasite exposures, has been associated with a cell-mediated response against the parasite's liver stage [19] and a required humoral response against the parasite's blood stage [20].

Currently there are no animal models for investigating the immunological impact of co-infection. Such a model would allow for control of confounding variables, such as multiple other infections, nutritional status, and ethical concerns, which could impact such studies in humans. To address the immunological impact of co-infection, we developed an Indian rhesus macaque co-infection model for HIV-1 and the relapsing malaria parasite *P. vivax* using SIV/Delta/B670 and *P. cynomolgi*, both established infection models for HIV [14], [18] and *P. vivax* [21] infections in rhesus macaques.

## Results

**Figure 1:**
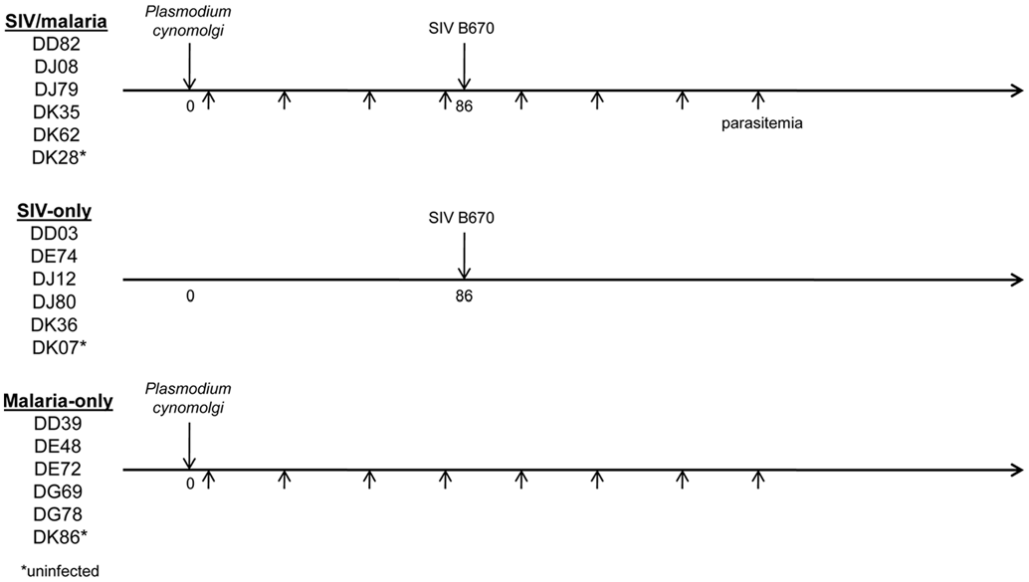
Experimental protocol. Eighteen rhesus macaques were randomly divided among three groups: SIV/malaria, SIV-only, and malaria-only. There were five experimental animals and one uninfected control animal (*) in each group. On day 0 the malaria parasite infected animals were inoculated with *Plasmodium cynomolgi*, and the SIV infected animals were inoculated with SIV/Delta/B670 following the third parasite relapse (day 86).

The pattern of co-infection we have chosen to model is adults living in regions of the world, such as Southeast Asia, that have a high incidence of new HIV-1 infections and are endemic for *P. vivax* malaria. Most individuals co-infected with HIV-1 and malarial parasites in these regions were exposed to *Plasmodium* before infection with HIV-1 as adults. To more closely model natural human exposure, co-infected animals were first inoculated with *P. cynomolgi* and then with SIV following the third parasite relapse for a total of four parasitemias (Figure 1). All parasite-infected animals were treated with chloroquine following parasite blood-stage emergence, which is necessary to avoid uncontrolled progression of the parasite infection and to avoid preventable distress or death of the animals. This treatment plan also more accurately models human malaria treatment in HIV positive individuals. All animals were closely monitored, and SIV-infected animals were humanely sacrificed following progression to Acquired Immune Deficiency Syndrome (AIDS).

Animals infected with *P. cynomolgi* in both the singly infected and co-infected groups displayed the expected malaria disease course with relapses occurring approximately once a month. Due to logistical reasons, the malaria-only group was inoculated at a later date than the SIV-infected animals. As a result the co-infected animals received a somewhat greater number of sporozoites due to decreased sporozoite yield when preparing the inoculums for the malaria-only animals. Since the number of parasite relapses is directly related to the number of sporozoites inoculated [22], the malaria-only animals did have fewer relapses than the co-infected animals. Additionally, the co-infected animals continued to relapse throughout the entire course of the SIV infection. While the difference in sporozoite number might have an impact during the erythrocytic stage, the chloroquine treatment following parasite appearance in the blood should have minimized such an effect by limiting parasitemias among the animals.

### Increased risk of disease progression in co-infected animals

**Figure 2:**
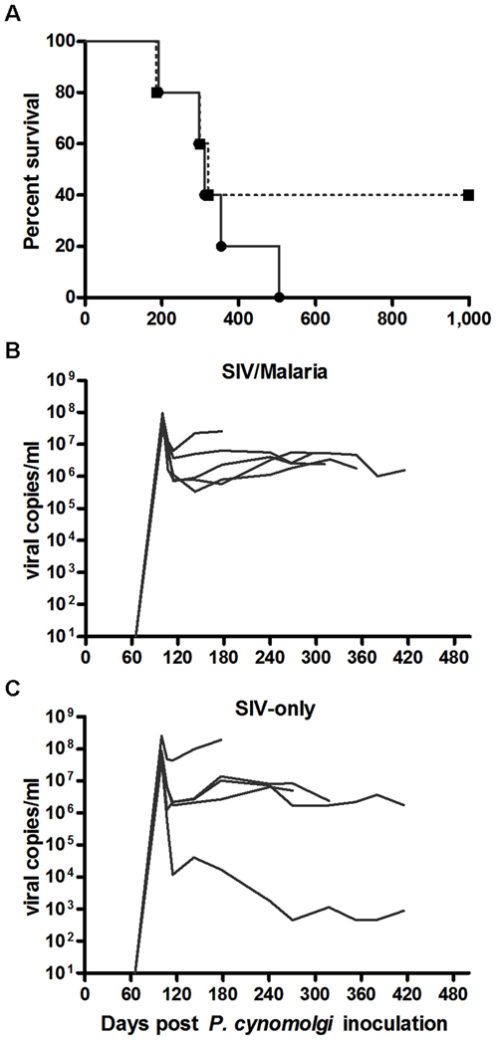
Decreased survival among co-infected animals was not associated with increased viral load. (A) Kaplan-Meier curve showing survival in the co-infected group (solid line; 1657 group survival days) and SIV-only group (dashed line; 2657 group survival days). Individual rhesus plasma viral RNA levels are shown for (B) co-infected and (C) SIV-only groups. Averaged viral set points (day 114) were 2.57×10^6^ and 9.95×10^6^ RNA copies/ml plasma, respectively.

There is much evidence from HIV/*P. falciparum* co-infection studies suggesting a negative outcome in co-infected individuals. From the present study, co-infection appears to increase the risk of SIV disease progression. All animals in the co-infected group progressed to AIDS while three out of five of the SIV-only animals progressed to AIDS during the three-year period of observation (Kaplan-Meier survival curve, Figure 2A). While the median animal of each group died at about the same time, the co-infected group survived a combined 1657 days while the SIV-only group survived a combined 2657 days with two animals in the SIV-only group surviving to the end of the study without progressing to AIDS. Predicting disease progression in SIV-infected macaques is not possible currently. However, it is possible that the *Plasmodium* co-infection could have converted conventional SIV progressors into more rapid progressors.

The co-infected group had a somewhat lower viral set point when compared with the SIV-only group (2.57×10^6^ vs. 9.95×10^6^ at day 114, respectively; Figure 2B and C) with each group having a rapid progressor that failed to control viral load. Therefore, the increased risk of disease progression among co-infected animals could not be attributed, in this case, to an increase in SIV viral load. This is in contrast to human studies describing transient increases in HIV viral load during parasitemia [11], [12]. The decreased SIV load is probably the result of chloroquine treatment which is known do decrease viral load in HIV infections [23]. Our treatment of the *Plasmodium*-infected animals could have artificially lowered SIV viral load, which will be investigated in future studies.

### Modulation of CD4+ and CD8+ T cells occurs during *P. cynomolgi* parasitemia, while co-infection accelerates CD4+ T cell decline

**Figure 3:**
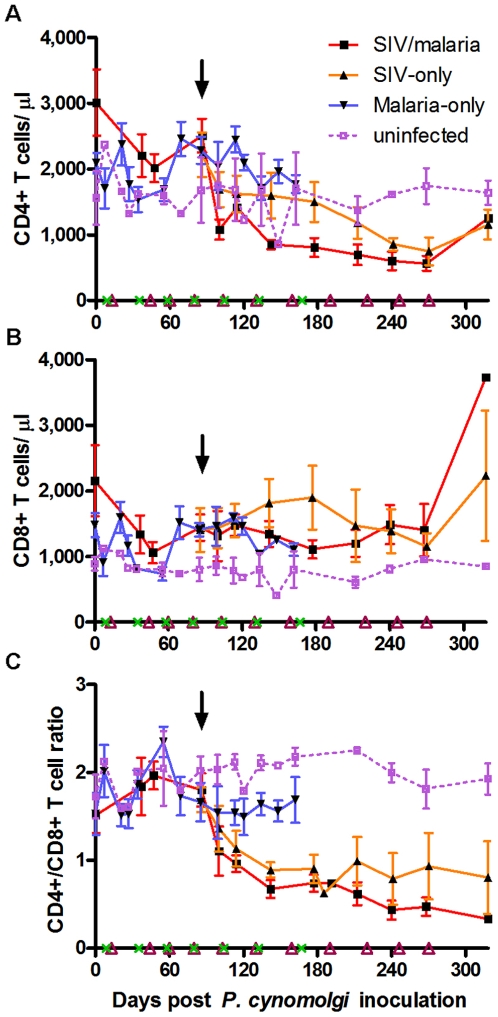
Impact of co-infection on CD4+ and CD8+ T cell dynamics and CD4+/CD8+ T cell ratios. Four-color flow cytometry showing T cell dynamics for (A) CD4+ T cells (CD3+CD20−CD4+CD8−), (B) CD8+ T cells (CD3+CD20−CD4−CD8+), and (C) CD4+/CD8+ T cell ratios. In this and subsequent figures, results from animals were averaged through day 318. The downward arrow indicates time of SIV inoculation (day 86). Parasitemias are indicated on the x-axis by a green x (malaria-only) or an open purple triangle (SIV/malaria). SIV-only animals were sampled starting at SIV infection, and the malaria-only animals were sampled until the group stopped relapsing. Results for individual animals throughout the course of infection are shown in Figure S1.

CD4+ T cells are required for the development and maintenance of a CD8+ T cell response [24], [25] as well as the optimal activation and development of the B cell antibody response [26]. To model a proliferative immune response to infection, four-color flow cytometry was utilized to follow changes in B cells and T cell subsets over the course of the infections (Table 1). In the figures displayed and in the statistical analysis performed, data from animals in each cohort were grouped through day 318 post *P. cynomolgi* infection. After day 318 the animal numbers declined rapidly from AIDS progression. For statistical analysis the malaria-only animals were compared to the uninfected control animals, and the co-infected animals were compared to the SIV-only animals following SIV infection through day 318. Dynamics of lymphocyte changes for individual animal are shown throughout the entire disease course in the Supplementary Figures S1 and S2. A characteristic immune response was observed among the malaria-only animals. CD4+ and CD8+ T cells (Figure 3A and B) increased in number in response to parasitemias, with each population having a sustained increase following the third parasitemia. CD4+/CD8+ T cell ratios fluctuated with the initial parasitemias but stabilized following the third (Figure 3C).

**Table 1:** Lymphocyte subsets based on cell surface markers as determined by four-color flow cytometry.

	Cell Marker
T cell subtype	FITC	PE	APC	PerCP
**CD4+ T cells**	CD3+	CD20−	CD4+	CD8−
**CD8+ T cells**	CD3+	CD20−	CD4−	CD8+
**B cells**	CD3−	CD20+		
**central memory CD4+ T cells**	CD95+	CD28+	CD3+	CD8−
**effector memory CD4+ T cells**	CD95+	CD28−	CD3+	CD8−
**naïve CD4+ T cells**	CD95−	CD29+	CD3+	CD8−
**CCR5+ memory CD4+ T cells**	CD45RA−	CCR5+	CD3+	CD8−

Following the fourth parasitemia, an adaptive immune response against *Plasmodium* would have been well developed. We wanted to address the question about SIV's immunosuppressive effects on the developed anti-malarial immune response and the effects of parasite-specific immune activation on SIV progression. After the third relapse, all SIV-infected animals were inoculated with SIV/Delta/B670. The SIV-infected animals had characteristic peripheral CD4+ T cell depletion (Figure 3A), but this decline was more than twice as rapid in the co-infected group than in the SIV-only group (regression analysis: slope for SIV/malaria = −3.30, SIV-only = −1.35; p = 0.028). The CD4+ T cells of the co-infected group increased in number in response to parasitemia prior to SIV inoculation, responded slightly to the subsequent parasitemia following SIV infection just before day 120, and failed to respond to the following parasitemias. This would suggest a diminished ability to immunologically respond to parasite exposures, supporting what was observed as an increased risk of infection and development of clinical malaria in human co-infection studies [3]–[10]. All co-infected animals responded to chloroquine treatment by clearing the parasite blood-stage throughout the study (data not shown).

Following SIV infection the CD8+ T cells of the SIV-only group significantly expanded over time (Figure 3B) when compared to the control animals (regression analysis: SIV-only = 2.01; uninfected animals = 0.09; p = 0.003). The CD8+ T cells of the co-infected animals initially declined (days 120–200, Figure 2B), but the interaction over time was not significant when compared to the SIV-only group. All SIV-infected animals had a decline in CD4+/CD8+ T cell ratios (Figure 3C), but this decline was nearly twice as rapid in the co-infected group than in the SIV-only group (regression analysis: SIV/malaria = −0.0029. SIV-only group = −0.0016; p = 0.009). This more rapid decline in CD4+ T cells and CD4+/CD8+ T cell ratios, both markers for HIV and SIV progression, supports the more rapid SIV progression observed among the co-infected animals.

### Co-infection alters T-cell subset dynamics

**Figure 4:**
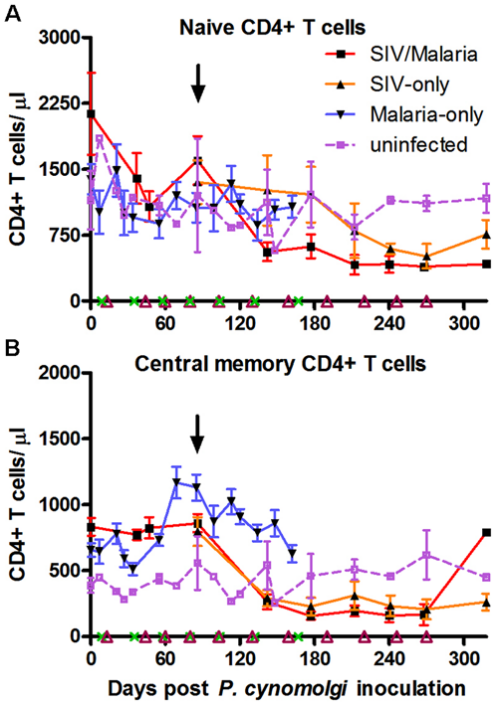
CD4+ T cell subset dynamics in SIV-infected, *P. cynomolgi*-infected and co-infected rhesus macaques. Four-color flow cytometry was used to follow group changes in CD4+ T cell subtypes (CD3+CD8−) over the course of infections for (A) naïve (CD95−CD28+) and (B) central memory (CD95+CD28+) CD4+ cells. Results for individual animals are shown in Figure S2.

To further characterize T cell subset dynamics during both infections, naïve (CD95-CD28+), central memory (CD95+CD28+), and activated memory (CD45RA−CCR5+) CD4+ T cells were quantified by four-color flow cytometry. Naïve T cells respond to pathogens that an individual's immune system has not yet encountered and replenishes lost memory CD4+ T cells, such as those lost due to HIV infection. This decrease in naïve CD4+ T cells has been associated, in non-rapid progressors, to SIV disease progression [15]. The loss of activated (SIV-specific and parasite-specific) memory CD4+ T cells by the SIV infection would increase pressure on replenishing the lost memory CD4+ T cells, increasing the naïve CD4+ T cell pool depletion and driving SIV progression. The numbers of naïve CD4+ T cells fluctuated in response to the parasite blood stage during relapses (malaria-only group; Figure 4A). Following SIV infection naïve CD4+ T cells were depleted more rapidly among the co-infected animals than among the SIV-only animals [regression analysis; SIV-only slope = −1.23, SIV-malaria slope = −2.77, p = 0.048; (Figure 4A)].

**Figure 5:**
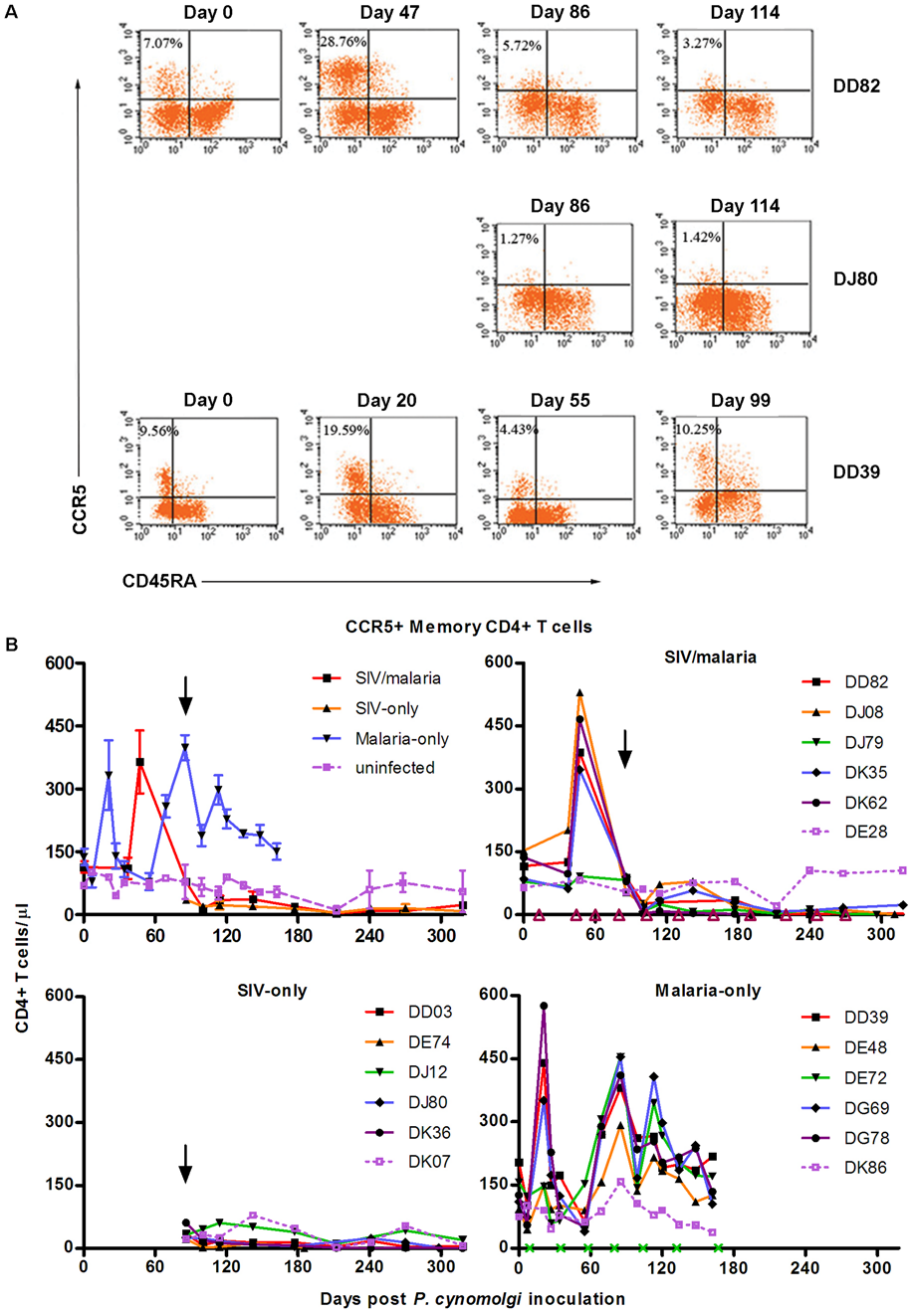
*Plasmodium* infection increases CCR5+ memory CD4+ T cell populations, which SIV infection rapidly eliminates. Four color flow cytometry was used to determine the CCR5+ memory CD4+ T cell populations (CD3+CD8−CD45RA−). (A) Representative results are shown for animals DD82 (SIV/malaria), DJ80 (SIV-only), and DD39 (malaria-only). (B) Averaged results are depicted with individuals in each group including the grouped uninfected animal.

Protective memory, such as antibody production and Th1/Th2 T cell and CD8+ T cell effector functions, are mediated by tissue-homing effector memory T cells. Central memory (CM) T cells migrate to secondary lymphoid organs in order to respond to subsequent antigen exposure [27]. CM CD4+ T cells (Figure 4B) and CCR5+ memory CD4+ T cells (Figure 5) proliferatively responded to parasitemia, presumably developing the adaptive immune response against the malaria parasite. Representative flow cytometry results of an animal in each group (CCR5+ memory CD4+ T cells) are shown in Figure 5A, and individual animals are shown over time in Figure 5B. CM CD4+ T cells were depleted following SIV infection among all SIV-infected animals (Figure 4B), and the co-infected animals failed to generate a CM CD4+ T cell response to parasitemias as observed among the malaria-only animals except for the longest surviving co-infected animal (DK36; Figure S2A). CCR5+ memory CD4+ T cells dramatically responded to parasitemias among the co-infected animals in a similar manner as the malaria-only animals (Figure 5C). Following SIV infection, this population was rapidly depleted and failed to respond to subsequent parasitemias.

### Anti-*Plasmodium* and anti-SIV antibody responses are altered in co-infected animals

**Figure 6:**
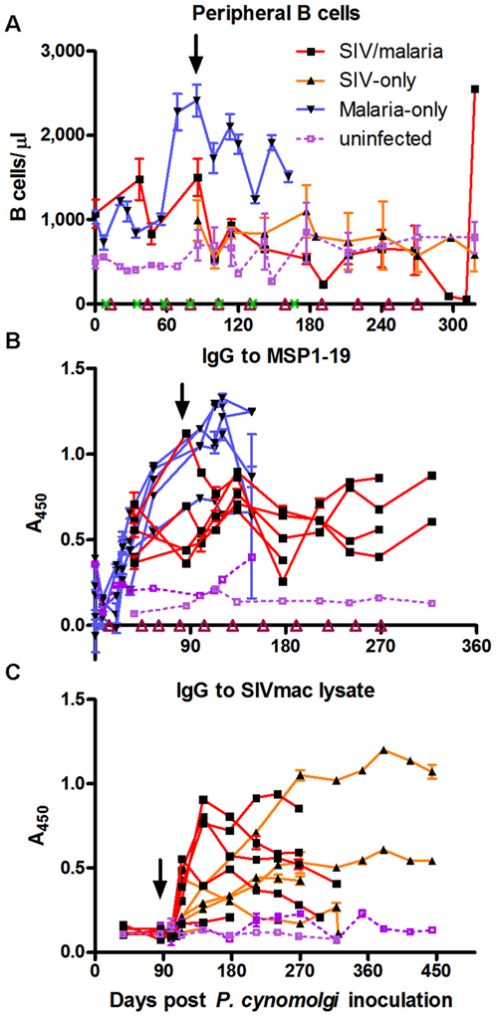
B cell response and altered IgG responses to *Plasmodium* and SIV among co-infected animals. (A) Peripheral B cells (CD3−CD20+) were followed by two-color flow cytometry. Serum was used to determine the (B) anti-MSP1-19 IgG host response and (C) anti-SIVmac lysate IgG response. Results using samples from individual animals are shown in panels (B) and (C), and individual B cell dynamics are shown throughout the course of infection in Figure S1.

B cells are an essential component of the adaptive immune system that has the principal function of producing specific antibodies against antigens of invading pathogens. Prior to SIV infection, B cells of the animals in the co-infected group initially responded to parasitemias in a similar manner as the malaria-only group (Figure 6A). Following SIV infection there was a slight B cell expansion, similar to the co-infected animals' CD4+ T cell response, following the subsequent parasitemia, but there was little or no proliferative response to the following parasitemias (Figure 6A). Except for the longest co-infected survivor, which had a similar but delayed B cell proliferation response as the malaria-only animals, all of the co-infected animals' B cell counts progressively declined to a significant level of depletion as the SIV infection progressed (Figure S1).

To profile the B cell functional response against both SIV and *Plasmodium* during co-infection, enzyme-linked immunosorbent assays (ELISAs) were used to monitor changes in IgG levels against an SIV viral lysate and the parasite protein MSP1-19, a highly antigenic blood-stage protein. The serum IgG response to the recombinant malaria parasite MSP1-19 protein by ELISA increased following sporozoite inoculation but decreased following SIV infection, continuing to fluctuate but not reaching the same magnitude as among the animals of the malaria-only group (Figure 6B). At the time of SIV infection, though, the B cell count was much greater among the malaria-only animals than among the co-infected animals, and this could have impacted the magnitude of the anti-parasite IgG response. The serum IgG response to the SIVmac viral lysate antigens increased more rapidly (peaked at day 28–56 post SIV inoculation) in the co-infected group than in the SIV-only group, as measured by absorbance (Figure 6C) and by endpoint titer (data not shown), and then declined. The IgG response among the SIV-only animals gradually increased to a plateau. The two rapid progressors, one in each of the SIV-infected groups, failed to generate an anti-SIVmac IgG response that was significantly different than that of the uninfected control animals (Figure 6C).

## Discussion

The results of this study show that the most widely used non-human primate models for HIV [14], [28]–[36] and *Plasmodium vivax* [21], [37]–[40] infections in humans can be successfully combined. Several consequences of *P. cynomolgi* and SIV co-infection of rhesus macaques were observed, including an increased risk of SIV disease progression and accelerated CD4 T-cell decline. Modulation of CD4+ and CD8+ T cells occurs during *P. cynomolgi* parasitemia, and co-infection alters these T-cell subset dynamics. Anti-*Plasmodium* and anti-SIV antibody responses were also altered in co-infected animals. These findings support previous reports of deleterious consequences of HIV/*Plasmodium* co-infections in humans [3]–[13] and establish an animal model system to further study the immunological and biological consequences of co-infection with these important pathogens.

Recent studies have found that, among conventional SIV progressors, the naïve CD4+ T cell pool is progressively depleted, most likely due to constant turnover of memory CD4+ T cells [15]. This suggests that there is replenishment of the memory CD4+ T cell pool by the naïve T cell pool, eventually leading to depletion of the naïve T cell pool and immune system collapse. A similar trend is observed in the co-infected animals in the present study in which there is a more rapid decline in the naïve CD4+ T cell pool. The generation of CCR5+ memory and central memory CD4+ T cells in response to the malaria parasite would be opportune targets for SIV infection [41]. Activation of parasite-specific CD4+ T cells, especially the CCR5+ CD4+ T cells, in response to parasitemia could be driving the higher viral loads as has been observed in HIV/*Plasmodium* co-infections [11], [12]. We did not observe an increased SIV load in co-infected animals; however, it is likely that this result was affected by the treatment of parasitic animals with chloroquine. Valuable information on the influences of co-infection on viral load could be gained in future studies by limiting chloroquine treatment following SIV infection while closely monitoring parasite levels in co-infected animals. Additionally, it is possible that species-specific factors could be what is driving the higher viral loads observed in co-infected humans.

A robust anti-parasite immunological response was rapidly generated in rhesus macaques infected with the parasite alone, and the numbers of circulating CD4+ and CD8+ T cells increased in response to parasitemia. Of the CD4+ T cell subtypes examined, activated CCR5+ memory CD4+ T cells strongly proliferated in response to parasitemia as well as central memory CD4+ T cells which are generated to respond to subsequent infections. B cells also proliferated in response to parasitemia, and a strong IgG response to one of the immunodominant surface proteins (MSP1-19) was observed. In the immediate period following co-infection with SIV, there was a slight proliferative response by CD4+ T cells and memory CCR5+ CD4+ T cells to the following parasitemia. However, subsequently there was either a failure of the proliferative response or rapid depletion of CD4+ T cells responding to parasitemia.

The proliferative B cell responses and the associated anti-parasite IgG response among the co-infected animals failed to reach the same magnitude as the animals infected with the parasite alone. While there is a difference in the numbers of B cells at the time of the SIV infection, which could have an impact on the magnitude of the anti-parasite B cell antibody response, the B cells among the co-infected animals fail to generate a significant proliferative response to parasitemias following SIV infection. This declining B cell population and the lack of a proliferative B cell response to parasitemia could possibly be due to inadequate CD4+ T cell help, either by central memory or newly activated parasite-specific naïve CD4+ T cells. This altered antibody response could have an impact on the parasite infection as a successful antibody response is required for parasite clearance [20], [42], [43]. In contrast, an anti-SIV antibody response was generated more rapidly in the co-infected animals and then declined. While in animals infected with SIV alone, the anti-SIV antibody response gradually increased and plateaus. It is possible that immune activation from the parasite infection primed the immune response to SIV. Additional analysis of CD4+ T cell subsets as well as CD8+ T cell subsets in the rhesus co-infection model could further define the loss of parasite-specific and SIV-specific effector functions.

Future studies with larger numbers of animals will be necessary to further characterize the SIV/malaria parasite co-infection model piloted here. More frequent immunophenotyping of lymphocytes during the co-infection will be needed to better profile proliferation changes in response to parasitemia. Quantifying changes in antigen-specific responses among CD4+ and CD8+ T cells longitudinally will help to determine if the SIV infection does indeed result in a loss of antigen recall to the malaria parasite. This potential loss of antigen recall could lower the threshold for developing clinical malaria when an HIV-infected individual or a pregnant woman is repeatedly infected with *Plasmodium*. Additionally, the immune activation associated with *Plasmodium* infection could lower the threshold for HIV infection. As we have demonstrated here, there is an increase in the numbers of HIV-receptive CCR5+ CD4+ T cells in response to parasitemia. Proinflammatory cytokines such as TNF-α, IL-1, and IL-6, which are upregulated during malaria, increase HIV proviral expression through the provirus modulatory enhancer region (reviewed in [41]). Such chronic inflammation associated with repeated *Plasmodia* and other infections could enhance HIV progression and spread. This co-infection model would be ideal for evaluating these situations.

Several combinations of SIV strains and *Plasmodia* species will eventually need to be examined to model various aspects of HIV-1 and *Plasmodium* co-infection. The fulminant disease induced by *P. knowlesi* in rhesus macaques more closely resembles severe *P. falciparum* malaria than malaria generally induced by *P. cynomolgi* or *P. vivax*. The form of malaria associated with the highest mortality is *P. falciparum* induced cerebral malaria. There are data to suggest that infections by certain simian *Plasmodia* species (e.g. *P. knowlesi*, *P. coatneyi*, or *P. fragile* in the rhesus macaque or other primates) can mimic some aspects of the pathobiology of human cerebral malaria [44]–[47]. The population that experiences cerebral malaria is comprised mostly of children below 6 years of age. Children are usually only infected with HIV congenitally or through breast-feeding. Here, we focused on a model of HIV and *Plasmodium* co-infection in adults in which exposure to the parasite usually occurs prior to infection with the lentivirus. Our group does have experience with the *P. coatneyi*/rhesus macaque model of malaria in pregnancy [48], [49], and has also studied congenitally acquired SIV infections [30], [36]. Therefore, we are well-positioned to adapt these models in future studies to address specific questions related to the impact of co-infection during pregnancy.

## Materials and Methods

### Ethics Statement

All animals were housed and handled at the Tulane National Primate Research Center (TNPRC) in strict accordance with the Guide for Care and Use of Laboratory Animals, and all animal protocols were approved by TNPRC Institutional Animal Care and Use Committee. TNPRC also adheres to the principles of humane animal experimentation proposed in *The Principles of Humane Experimental Technique* (1959). All parasite-infected animals were monitored without anesthesia for parasitemia, and the blood-stage infection was treated with chloroquine. All SIV infected animals were under close veterinary observation and, following the progression to AIDS, were humanely euthanized.

### Animals

Ten Indian rhesus macaques were inoculated intravenously with either 4,000 (malaria-only) or 12,000 (SIV/malaria) sporozoites. Following the third parasite relapse, ten animals (SIV/malaria and SIV-only) were inoculated intravenously with SIV/Delta/B670. All parasite-infected animals were monitored daily without anesthesia for parasitemia, and the blood-stage infection was treated with chloroquine (7 mg/kg/day for five days).

### SIV and virus quantification

The SIV/Delta/B670, a highly pathogenic virus, has been developed and used at the TNPRC [18], and had been previously prepared in primary rhesus peripheral blood mononuclear cells. Following the third parasite relapse, ten animals (SIV/malaria and SIV-only) were inoculated intravenously with SIV/Delta/B670. Viral load was determined by branched-DNA analysis using EDTA plasma (Bayer Diagnostics, Inc.).

### Sporozoite preparation and infection

A splenectomized, adult rhesus macaque was inoculated in the saphenous vein with approximately 10^6^ previously frozen parasitized red blood cells to initiate a blood stage infection. When gametocytes first appeared in the blood, *Anopheles stephensi* mosquitoes from the mosquito colony at TNPRC were fed (300 per day) on the anesthetized donor animal. Following this cycle the donor animal was treated with a curative dose of chloroquine (7 mg/kg/day) for five days. Infected mosquitoes were maintained at 27°C for two weeks, and sporozoites were dissected from the salivary glands for the experimental animal inoculation. Ten animals were inoculated intravenously with either 4,000 (malaria-only) or 12,000 (SIV/malaria) sporozoites; the difference in sporozoite numbers was a result of decreased sporozoite recovery for the malaria-only inoculum preparation. Additionally, all parasite-infected animals were treated with chloroquine when parasites appeared in the blood, minimizing possible impact of the differences in sporozoite number.

### Sample collection

Heparin and EDTA treated blood were collected 1) every 7 days for 28 days following SIV inoculation and every 30 days thereafter for the SIV-infected groups; or 2) every 7 days following sporozoite inoculation for 28 days and every 14 days thereafter.

### Lymphocyte immunophenotypic analysis

Peripheral lymphocyte counts were determined by an automated, complete blood count. Lymphocyte subset changes were followed by four-color flow cytometry. Briefly, 100 µl of EDTA treated whole blood was stained with CD3-APC, CD8-PerCP, and (i) CD45RA-FITC and CCR5-PE, (ii) CD95-FITC and CD28-PE, or CD4-APC, CD8-PerCP, CD3-FITC, and CD20-PE. All antibodies were obtained from BD Biosciences. Following whole blood lysis, stained lymphocytes were fixed in 1% paraformaldehyde, acquired on a FACSCalibur (BD Biosciences), and analyzed using CellQuest (BD Biosciences).

### Statistical analysis

Regression analyses were used to test the null hypotheses that 1) malaria parasite infection has no impact on the change of outcome compared to the uninfected control animals (from day 0), 2) SIV alone has no impact on the change in outcome between the control animals and SIV-only animals (from the SIV inoculation date of day 86 to day 318), and 3) malaria plus SIV has no impact on change in outcome between SIV-only and SIV/malaria animals (from day 86). These analyses take into account initial differences in analyzed values among animals while examining rates of change. A linear relationship between time and outcome was assumed, and generalized estimating equations were used to estimate the parameters and their standard errors.

### Immunoassays

Rhesus IgG responses to a recombinant *P. vivax* malaria parasite protein MSP1-19 [yP.v.200 MSP1-19 2905/6 (MRA-47, MR4, ATCC Manassas Virginia)] [50] were determined by enzyme-linked immunosorbent assay (ELISA). 96-well ELISA plates [low volume, high binding ELISA plates (Corning, Inc.)] were coated with 10 ng/well of recombinant protein in coating buffer (0.1 M sodium bicarbonate, pH 9.2) overnight at 4°C. Plates were washed with wash buffer [PBS with 0.5% Tween 20 (Sigma-Aldrich)], blocked for one hour with StartingBlock T20 PBS (Pierce Biotechnology), dried, and stored at 4°C until use. Sera were serially diluted, starting with a 1∶10 dilution, with carrier buffer (PBS supplemented with 10% StartingBlock T20 PBS), and 50 µl of diluted serum, in duplicate, was added to the 96-well plates and incubated at room temperature for one hour. Plates were washed (5×150 µl wash buffer), incubated with a biotinylated mouse anti-human IgG antibody [(BD Pharmingen); 1∶1000 dilution in carrier buffer], washed, and incubated for one hour at room temperature with an avidin-horseradish peroxidase antibody [(BD Pharmingen); 1∶1000 dilution in carrier buffer]. Plates were washed and incubated for 30 minutes with 50 µl substrate [1-step Turbo TMB-ELISA (Pierce Biotechnology)]. Reactions were stopped with 50 µl 1 M phosphoric acid and absorbance read at A_450_.

Rhesus IgG responses to SIV [SIVmac251 purified viral lysate (ZeptoMetrix, New York)] were determined by ELISA. 96-well plates were coated with 34.29 ng/well of SIV lysate for one hour at 37°C. Following a wash (1×200 ul wash buffer), plates were blocked for one hour with 4% milk [PBS with Blocking-grade blocker (Bio-Rad)], washed, and incubated overnight at 4°C with serum serially diluted 1∶4 starting at 1∶400 in PBS. Plates were washed and incubated with 50 µl of an anti-human IgG-horseradish peroxidase antibody [(BD Pharmingen); 1∶2000 dilution in wash buffer] for two hours at room temperature. Plates were washed (5×200 µl), incubated for 20 minutes with 50 µl substrate [1-step Turbo TMB-ELISA (Pierce Biotechnology)], the reactions were stopped with 50 µl 1 M phosphoric acid, and the absorbance was read at A_450_.

## Supporting Information

Figure S1 Individual animal responses to SIV and/or malaria parasite infection by group for peripheral lymphocytes. Flow cytometry was used to follow changes in peripheral CD4+ (CD20-CD3+CD4+) T cells (A), CD8+ (CD20-CD3+CD8+) T cells (B), and B (CD20+CD3-) cells (C). The malaria-only animals were followed until they stopped relapsing and are shown out to day 180 while the SIV-infected animals are shown out to day 630. Parasitemias are shown on the x-axis as an open triangle (SIV/malaria) or a green x (malaria-only), and SIV infection (day 86) is indicated with an arrow. The grouped control animal is shown with the purple dashed line.
(1.26 MB TIF)

Figure S2 Individual animal responses by group of naïve and central memory CD4+ T cells. Central memory (CD95+CD28+) CD4+ T cell (A) and naïve (CD95-CD28+) CD4+ T cell (B) dynamics in response to SIV and/or malaria parasite infection. The malaria-only animals were followed until they stopped relapsing and are shown out to day 180 while the SIV-infected animals are shown out to day 630. Parasitemias are shown on the x-axis as an open triangle (SIV/malaria) or a green x (malaria-only), and SIV infection (day 86) is indicated with an arrow. The grouped control animal is shown with the purple dashed line.
(0.85 MB TIF)
