# Supplementary figures and images for: Altered Immune Responses in Rhesus Macaques Co-Infected with SIV and Plasmodium cynomolgi: An Animal Model for Coincident AIDS and Relapsing Malaria
Source: PLoS One. 2009 Sep 23;4(9):e7139. doi: 10.1371/journal.pone.0007139 (PMC2744481; doi:10.1371/journal.pone.0007139)

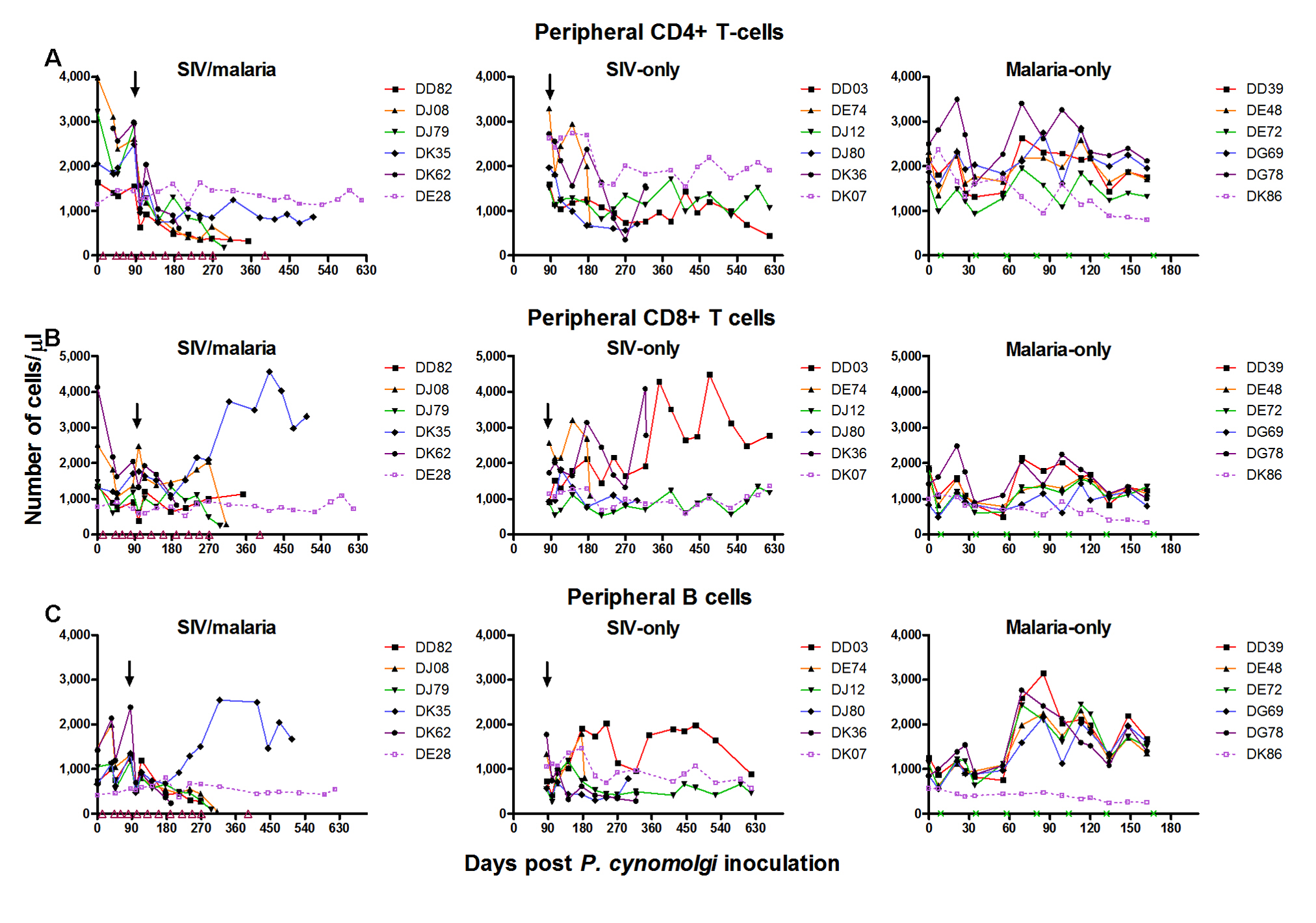

Supplement: Figure S1 — Individual animal responses to SIV and/or malaria parasite infection by group for peripheral lymphocytes. Flow cytometry was used to follow changes in peripheral CD4+ (CD20-CD3+CD4+) T cells (A), CD8+ (CD20-CD3+CD8+) T cells (B), and B (CD20+CD3-) cells (C). The malaria-only animals were followed until they stopped relapsing and are shown out to day 180 while the SIV-infected animals are shown out to day 630. Parasitemias are shown on the x-axis as an open triangle (SIV/malaria) or a green x (malaria-only), and SIV infection (day 86) is indicated with an arrow. The grouped control animal is shown with the purple dashed line. (1.26 MB TIF) [file pone.0007139.s001.tif]

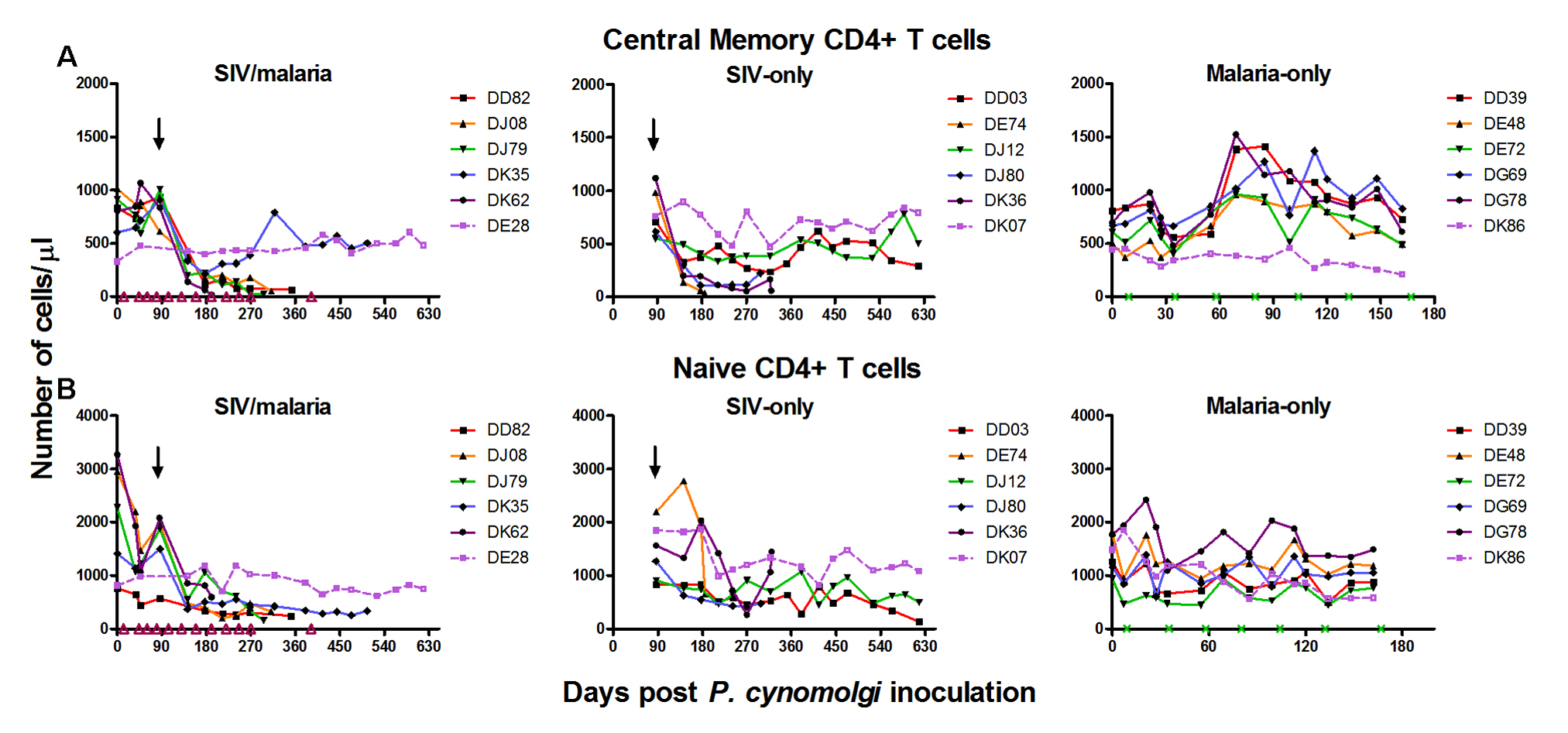

Supplement: Figure S2 — Individual animal responses by group of naïve and central memory CD4+ T cells. Central memory (CD95+CD28+) CD4+ T cell (A) and naïve (CD95-CD28+) CD4+ T cell (B) dynamics in response to SIV and/or malaria parasite infection. The malaria-only animals were followed until they stopped relapsing and are shown out to day 180 while the SIV-infected animals are shown out to day 630. Parasitemias are shown on the x-axis as an open triangle (SIV/malaria) or a green x (malaria-only), and SIV infection (day 86) is indicated with an arrow. The grouped control animal is shown with the purple dashed line. (0.85 MB TIF) [file pone.0007139.s002.tif]
